# survkl: an R package for transfer-learning-based integrated Cox models

**DOI:** 10.1093/bioadv/vbag234

**Published:** 2026-08-25

**Authors:** Yubo Shao, Lingfeng Luo, Xiaohan Liu, Junyi Qiu, Di Wang, Kevin He

**Affiliations:** Department of Biostatistics, University of Michigan School of Public Health, Ann Arbor, MI 48109, United States; Department of Biostatistics, University of Michigan School of Public Health, Ann Arbor, MI 48109, United States; Department of Biostatistics, University of Michigan School of Public Health, Ann Arbor, MI 48109, United States; Department of Biostatistics, University of Michigan School of Public Health, Ann Arbor, MI 48109, United States; Division of Biostatistics, Data Science Institute, Medical College of Wisconsin, Milwaukee, WI 53226, United States; Department of Biostatistics, University of Michigan School of Public Health, Ann Arbor, MI 48109, United States

## Abstract

**Summary:**

Survival risk prediction often suffers from challenges such as rare event rates, small effective sample sizes, high-dimensional feature spaces, weak signals, population heterogeneity, and concerns over patient privacy. To overcome these obstacles and improve the precision of prognostic modeling, we introduce the survkl software, which enables the incorporation of external summary-level information with newly collected time-to-event data to support more robust and accurate predictions in survival analysis. Our method adaptively adjusts the weight given to external information, down-weighting heterogeneous information and highlighting more informative ones. The proposed tool accommodates both low-dimensional and high-dimensional data, offering unpenalized estimation and computationally efficient lasso, ridge, and elastic net penalties. The survkl software also provides auxiliary evaluation and plotting functions for model assessment.

**Availability and implementation:**

survkl is freely available to the public at https://github.com/UM-KevinHe/survkl and published under General Public License version 3 license.

## 1 Introduction

Prognosis prediction is crucial in survival analysis. While the surge of biobank data (e.g. genetics, transcriptomics, Electronic Health Record (EHR)) offers great potential, it faces challenges like limited effective sample sizes, high dimensionality, low signal-to-noise ratios, and patient privacy concerns. Although integrating external information is expected to enhance prediction performance, classical integration approaches often fail due to the unrealistic assumption of similar underlying distributions across data sources. Thus, it is crucial to develop a transfer learning procedure that effectively accounts for population differences.

Although Kullback–Leibler (KL) divergence has been proposed to integrate external predicted probabilities for binary outcomes (Schapire *et al*. 2005, Jiang *et al*. 2016), such approaches are not directly applicable to censored time-to-event data. In survival analysis, censored outcomes provide only partial information: they indicate that the event of interest has not occurred up to the observed censoring time but do not reveal the actual failure time. This incomplete observation complicates the direct computation of probability distributions over failure times, which is required for applying KL divergence. Moreover, published risk scores for time-to-event data, such as polygenic hazard scores (Seibert *et al*. 2018), which are computed as weighted sums of single-nucleotide polymorphisms using effect sizes from Cox models (Cox 1972), typically include only covariate coefficients without baseline hazard information. As a result, although these risk scores remain clinically valuable, their lack of a direct probabilistic interpretation prevents their use by existing integrated methods (Chen *et al*. 2021, Sheng *et al*. 2021, Cheng *et al*. 2023) that require a full probabilistic specification. More recently, transfer-learning approaches for survival data, including boosting-based (Bellot and van der Schaar 2019) and adaptive Cox (Lu *et al*. 2026) methods, require individual-level external data, which is often unavailable because of privacy constraints.

To address these issues, we previously proposed a partial likelihood KL-based transfer learning procedure (Wang *et al*. 2026) for integrating published regression coefficients with internal Cox models while addressing population heterogeneity. Despite this methodological progress, ready-to-use software implementing transfer-learning methods for survival data remains scarce, which limits their adoption in practice. Moreover, another common challenge arises from the use of point-based clinical prognostic tools, such as those developed for prostate cancer (Dess *et al*. 2020). These tools assign weights to a set of clinical factors to stratify patients into risk groups (e.g. low, intermediate, and high risk). Although useful for clinical decision making, such groupings are based solely on clinical knowledge, lack a probabilistic interpretation, and are not compatible with existing integration methods. To fill in these gaps, we introduce a transfer learning-based R package survkl for efficient survival data integration, leveraging external information alongside newly collected time-to-event data. This package offers the following key features: (i) it uses only summary-level external information, but with no use of any sensitive individual-level external data, ensuring patient privacy is protected, while requiring less external information than alternative summary-level methods that additionally need the external baseline hazard or second-order information; (ii) it is robust to model mis-specification and supports various formats of external summary information, including risk scores derived from external models and ranking-based on clinical risk groups, making it broadly applicable across diverse datasets; (iii) it controls the relative weight of different sources of external information, identifying and prioritizing the most compatible information while diminishing the influence of less relevant sources; (iv) it implements various machine learning procedures, including ridge regression (Hoerl and Kennard 1970), LASSO (Tibshirani 1997), and Elastic Net (Simon *et al*. 2011), thereby effectively addressing high dimensionality; and (v) it accommodates that the external study may only include partial information on a subset of predictors in the internal study.

## 2 Methods

### 2.1 Cox proportional hazards model for the target cohort

Let $D_{i}$ denote the death time and $C_{i}$ be the censoring time for patient *i*, i=1,…,n, where *n* is the total sample size of the target cohort. The observed survival time is $T_{i}=\text{min}\{D_{i},C_{i}\}$, and the death indicator is given by $\delta_{i}=I(D_{i}\leq C_{i})$. Let $Z_{i}={\left(Z_{i1},\ldots ,Z_{ip}\right)}^{T}$ be a *p*-dimensional covariate vector for the *i*-th patient. We assume that, conditional on $Z_{i}$, $D_{i}$ is independently censored by $C_{i}$. Consider hazards $\lambda (t|Z_{i})=\lambda_{0}(t)\;\text{exp}\;\{g(Z_{i},\beta )\}$, where $\lambda_{0}(t)$ is an arbitrarily unspecified baseline hazard function, $g(Z_{i},\beta )$ specifies the log-relative risk relationship between the covariates $Z_{i}$ and the hazard function, and β is a vector of parameters. The log-partial likelihood is given by


(1) $$\ell (\beta )=\sum_{i=1}^{n}\delta_{i}\left[g(Z_{i},\beta )-\text{log}\;\left\{\sum_{l=1}^{n}Y_{l}(T_{i})\;\text{exp}\;\{g(Z_{l},\beta )\}\right\}\right],$$

where $Y_{l}(T_{i})=I(T_{l}\geq T_{i})$ is the at-risk indicator.

### 2.2 External summary information

To account for privacy constraints, we consider scenarios where only external summary information is available, rather than individual-level external data. For example, suppose the estimated coefficients $\tilde{\beta }$ are available from a published Cox model; a risk score can be calculated as $\tilde{g}(Z_{i})=Z_{i}^{⊤}\tilde{\beta }$ for the *i*-th subject in the target cohort. The proposed transfer learning procedure is flexible and can incorporate various forms of external summary information, including estimated risk scores from machine learning algorithms and clinically derived risk groupings. Because the procedure requires only a relative-risk score for each subject, the external model may take essentially any form—a fitted survival model, a machine-learning risk predictor, or a clinical risk grouping—without supplying an external baseline hazard or a calibrated survival probability.

### 2.3 Partial likelihood-based transfer learning

To extract information from external risk scores, we formulate the censored time-to-event data as a dynamic ranking problem. Specifically, suppose the internal cohort comprises *K* unique failure times $t_{1}<\cdots <t_{K}$. Let $A_{k}$ specify that individual *k* fails in $\left[t_{k},t_{k}+dt_{k}\right)$. Let $B_{k}$ specify all the censoring and failure information up to time $t_{k}^{-}$ as well as the information that one failure occurs in $\left[t_{k},t_{k}+dt_{k}\right)$. Based on the external risk scores, the conditional density of $A_{k}$ given $B_{k}$ is


(2) $$\begin{array}{c} \tilde{f}(A_{k}|B_{k})=\frac{{\tilde{\lambda }}_{0}(t_{k})\;\text{exp}\;\{\tilde{g}(Z_{k})\}dt_{k}}{\sum_{i=1}^{n}Y_{i}(t_{k}){\tilde{\lambda }}_{0}(t_{k})\;\text{exp}\;\{\tilde{g}(Z_{i})\}dt_{k}}\\ \;=\frac{\;\text{exp}\;\{\tilde{g}(Z_{k})\}}{\sum_{i=1}^{n}Y_{i}(t_{k})\;\text{exp}\;\{\tilde{g}(Z_{i})\}}, \end{array}$$

where the second equality follows from canceling ${\tilde{\lambda }}_{0}(t_{k})dt_{k}$ in the numerator and the denominator. Following Wang *et al*. (2026), the partial likelihood-based KL divergence between conditional densities corresponding to the external risk scores and the internal Cox model, contained in $A_{k}|B_{k}$, is given by


(3) $$d_{KL}(\tilde{f}∥f;t_{k})=E_{\tilde{f}}\left[\text{log}\;\left\{\frac{\tilde{f}(A_{k}|B_{k})}{f(A_{k}|B_{k})}\right\}\right],$$

where the expectation is with respect to the conditional density $\tilde{f}(A_{k}|B_{k})$ corresponding to the external risk scores, and $f(A_{k}|B_{k})$ is the conditional density based on the internal Cox model,


(4) $$f(A_{k}|B_{k})=\frac{\;\text{exp}\;\{g(Z_{k},\beta )\}}{\sum_{i=1}^{n}Y_{i}(t_{k})\;\text{exp}\;\{g(Z_{i},\beta )\}}.$$

When $\tilde{g}(Z_{k})$ are generated from clinically derived risk groupings, $\tilde{f}(A_{k}|B_{k})$ in (2) does not represent a formal conditional density. Instead, it can be viewed as a Plackett-Luce (Plackett 1975) ranking metric. In such cases, $d_{KL}(\tilde{f}∥f;t_{k})$ in (3) can be interpreted as a generalized KL divergence. The accumulated KL divergence across the sequence of conditional experiments $A_{1}|B_{1},\ldots ,A_{K}|B_{K}$ is $D_{KL}(\tilde{f}∥f)=\sum_{k=1}^{K}d_{KL}(\tilde{f}∥f;t_{k}),$ which measures the discrepancy between external risk scores and the internal Cox model. To integrate external information while accounting for potential disparities, we combine the log-partial likelihood from the target cohort with the accumulated KL divergence by constructing a penalized objective function:


(5) $$\ell_{\eta }(\beta )=\ell (\beta )-\eta D_{KL}(\tilde{f}∥f),$$

where η is a tuning parameter that controls the trade-off between the internal model and the external risk scores. The detailed expression of $\ell_{\eta }(\beta )$ is provided in Section 1, available as supplementary data at *Bioinformatics Advances* online.

### 2.4 Regularization for high-dimensional data

For high-dimensional applications, we extend the objective function in (5) by adding a regularization term. The resulting objective function enables simultaneous variable selection and parameter estimation:


(6) $$\ell_{\eta ,\lambda }(\beta )=\ell_{\eta }(\beta )-\lambda P(\beta ),$$

where P(β) is a penalty function (further details are provided in the subsection below) and λ is a tuning parameter controlling its strength.

### 2.5 Implementation

The core functionality of the *survkl* package includes the *coxkl* function, which fits the integrated Cox model in low-dimensional settings. It flexibly accommodates external information supplied either as a vector of coefficient estimates or as pre-computed risk scores. For high dimensional data, the *coxkl_ridge* and *coxkl_enet* function extend the method by implementing ridge, LASSO, and elastic net penalties. Each fitting function is paired with a corresponding cross-validation function (*cv.coxkl*, *cv.coxkl_ridge* and *cv.coxkl_enet*), which performs K-fold cross-validation to select the optimal integration weight η and the regularization parameter λ. Evaluation metrics utilized include Harrell’s C-index (Harrell Jr *et al*. 1996) for discrimination and V&VH loss function (Verweij and van Houwelingen 1993) for overall model fit. Furthermore, the performance metrics and cross-validation results can be easily visualized using the *plot* and *cv.plot* to guide this process.

## 3 Example usage

### 3.1 Simulation

We simulate data under the Cox model $\lambda \left(t|Z\right)=2t\;\text{exp}(Z^{⊤}\beta )$, with true coefficients $\beta ={\left(0.3,-0.3,0.3,-0.3,0.3,-0.3\right)}^{⊤}$. Six covariates $Z={\left(Z_{1},\ldots ,Z_{6}\right)}^{⊤}$ are defined as follows: ${\left(Z_{1},Z_{2}\right)}^{⊤}$ are jointly Gaussian with zero mean, unit variances, and an AR(1) correlation structure (correlation parameter ρ=0.5); $Z_{3}$ and $Z_{4}$ are independent Bernoulli variables with success probability 0.5; $Z_{5}$ and $Z_{6}$ are unit-variance normal variables with means $2L_{i}$ and $-2L_{i}$, where the latent group $L_{i}$ follows $\text{Bernoulli}(p_{L})$. For internal datasets we set $p_{L}=1$ and impose independent right censoring to achieve a censoring rate of 0.70. In each of the 500 replications, we generate a training set (n=100) and a matching test set (n=2000). For external information, we use three quality-specific external cohorts (Good/Fair/Poor; n=2000, censoring rate 0.50) and fit corresponding external models. External quality is controlled jointly by the latent group prevalence in the external data ($p_{L}=1.0/0.5/0.0$ for Good/Fair/Poor) and by the number of covariates used to train the external model (Good: $Z_{1}$–$Z_{6}$; Fair: $\left\{Z_{1},Z_{3},Z_{5},Z_{6}\right\}$; Poor: $\left\{Z_{1},Z_{5}\right\}$), yielding decreasing concordance with the internal truth.

**Figure 1:**
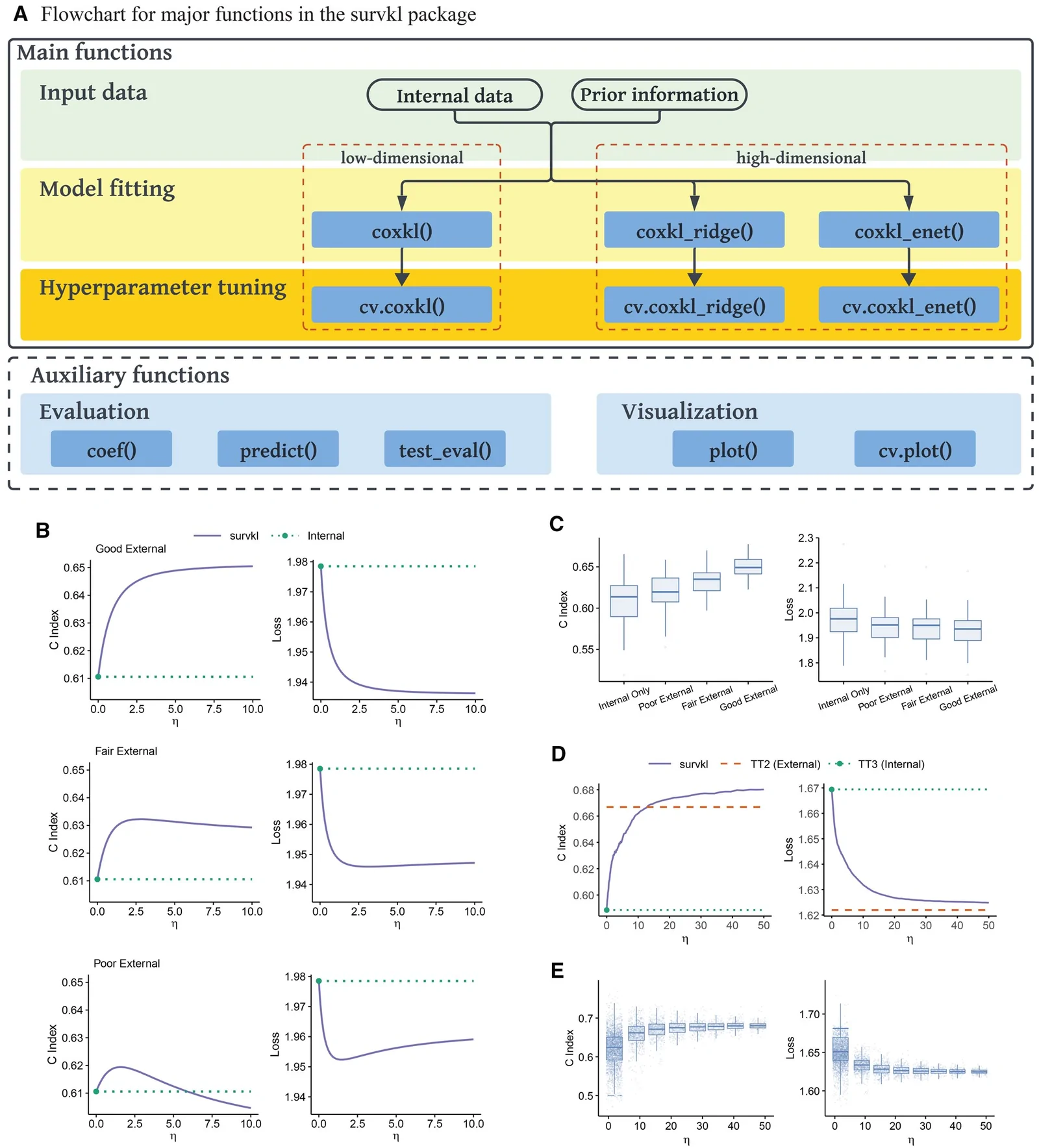
(A) Flowchart illustrating the major functions in the survkl package. Low-dimensional data are fitted using coxkl for the integrated model and cv.coxkl for cross-validation to select the optimal integration parameter. High-dimensional data are fitted using coxkl_ridge (ridge penalty) or coxkl_enet (elastic net or LASSO penalty), with cv.coxkl_ridge and cv.coxkl_enet used for hyperparameter tuning. (B) 500 times average test-set performance of the proposed integrated method (implemented by the coxkl function) across prior weights η under Good/Fair/Poor external models. Good External yields monotone gains as η increases; Fair shows a non-monotone trade-off; even Poor provides a small-η improvement, while large η causes negative transfer. (C) Boxplots over 500 replications comparing Internal Only with the proposed integrated method (implemented by the coxkl function) using Poor/Fair/Good external models (η chosen by cross-validation). Higher external quality raises the median C-index and lowers loss with less variability; even Poor outperforms Internal Only on average. (D) Test-set performance of the proposed integrated model with an LASSO penalty under varying prior weights (η) from the TT2 cohort. As η increases, borrowing information from TT2 enhances model discrimination and reduces loss, with the integrated model outperforming both the internal and external models across a range of η values. (E) Robustness assessment of the integrated model based on 500 random train–test splits of the TT3 cohort. The boxplots display the distribution of model performance across varying η values. As more prior information from TT2 is incorporated, estimation variability decreases and the median C-index and loss improved.

Figure 1B reports 500 times average performance of the proposed integrated method across KL weights η, which also includes the internal-only baseline. With Good external information, the C-index (higher is better) increases sharply before leveling off. Meanwhile, the loss (lower is better) steadily decreases and approaches a limit. This shows that gaining external information leads to steady improvements. With Fair quality, both metrics are non-monotone: performance improves for moderate η and then slightly declines. This reflects a trade-off between bias and variance when the external model is partially misestimated. Even with poor quality, there exists a beneficial window at small η that the C-index is higher than the internal baseline, and the loss reaches its lowest point before larger η induces negative transfer. Overall, external information can still be useful across all quality levels. Data adaptive tuning (e.g. cross-validation) picks small η under Poor, moderate η under Fair, and larger η under Good, resulting in consistent improvements while avoiding excessive borrowing. Figure 1C compares performance across four strategies: Internal Only and integration with Poor, Fair, and Good external models. As the external quality improves, the median C-index goes up and the interquartile range narrows. At the same time, the loss goes down, with fewer high-loss outliers. Importantly, even the Poor External condition performs better than the Internal Only model on average. This shows that borrowing can extract a useful signal despite some mismatch. The gains become larger and more stable for the Fair and Good External models.

### 3.2 Application

We analyze gene expression and survival outcomes from multiple myeloma patients enrolled in the University of Arkansas for Medical Sciences clinical trials, which studied Total Therapy II (TT2) and Total Therapy III (TT3), respectively. These datasets are publicly available through the MicroArray Quality Control Consortium II (MAQC-II) study (Shi *et al*. 2010), under the GEO accession number GSE24080. Gene expression profiling was performed using the Affymetrix Human Genome U133 Plus 2.0 microarrays. We use the TT3 cohort (n=213, with 55 deaths and 20 502 gene-level covariates) as the internal dataset to model survival outcomes, while incorporating prior information from the TT2 cohort (n=339, with 190 deaths) as an external source. To evaluate model generalizability, we randomly partition the TT3 data into a training subset (60%) for model estimation and a held-out test subset (40%) for performance assessment. The overall survival time is defined as the duration from the date of diagnosis to either death or the last follow-up.

Figure 1D, generated by the cv.plot function, illustrates the performance of the proposed integrated model with an LASSO penalty under varying weights (η) assigned to the prior TT2 information. When no prior information is incorporated (η=0), the model fitted on TT3 data alone yields a C-index of approximately 0.58 and a loss of about 1.67, while the prior model trained on TT2 achieves a C-index of roughly 0.67 and a loss of 1.62 when evaluated on the TT3 test data. As the internal model gradually borrows information from the TT2 prior, there exists a range of η values where the integrated model achieves better risk discrimination of patient survival than both the TT3-based internal model and the TT2-based external model. Figure 1E further evaluates the robustness of the proposed model through 500 random train–test splits of the internal TT3 data. The boxplots depict the distribution of model performance across different values of η. As the model borrows more external information from TT2, the estimation variance gradually decreases, and the median values of both the C-index and loss improve.

## 4 Discussion

To integrate published survival models with newly collected time-to-event data, we develop a transfer learning-based R package, survkl, which accounts for heterogeneity across data sources by identifying the most compatible datasets and down-weighting less relevant ones. In addition, the proposed tool flexibly incorporates various types of external prediction models and achieves computational efficiency when paired with a range of machine learning algorithms.

## Supplementary Material

vbag234_Supplementary_Data

## Data Availability

The survkl package is freely available at https://github.com/UM-KevinHe/survkl.
