# Supplementary material for: survkl: an R package for transfer-learning-based integrated Cox models
Source: Bioinform Adv. 2026 Aug 25;6(1):vbag234. doi: 10.1093/bioadv/vbag234 (PMC13528772; doi:10.1093/bioadv/vbag234)
Supplement: Supplementary file 1 [file vbag234_supplementary_data.pdf]

Supplementary Material

survkl: an R package for transfer-learning-based integrated Cox models

Yubo Shao, Lingfeng Luo, Xiaohan Liu, Junyi Qiu, Di Wang, Kevin He

S1 Supplementary Methods

S1.1 Closed-form expression of the integrated objective

The integrated objective in (5) combines the internal log-partial likelihood with the accumulated Kullback–Leibler (KL) divergence between the external and internal conditional densities. We show here that it admits a closed form that coincides with an ordinary Cox log-partial likelihood in which the event indicator is replaced by an externally informed pseudo-outcome. This closed form both explains why integrating the external information costs no more than a single Cox fit and motivates our choice of the divergence direction $D_{KL}(\tilde{f} \parallel f)$.

For each unique failure time t_k ($k = 1, \ldots, K$), let $A_k = j$ denote the event that subject j is the one failing among those at risk. Generalizing the conditional densities (2) and (4) to an arbitrary at-risk subject j, we write

$$\tilde{f}(A_k = j \mid B_k) = \frac{Y_j(t_k)\exp\{\tilde{g}(\mathbf{Z}_j)\}}{\sum_{i=1}^{n} Y_i(t_k)\exp\{\tilde{g}(\mathbf{Z}_i)\}}, \qquad f(A_k = j \mid B_k) = \frac{Y_j(t_k)\exp\{g(\mathbf{Z}_j, \boldsymbol{\beta})\}}{\sum_{i=1}^{n} Y_i(t_k)\exp\{g(\mathbf{Z}_i, \boldsymbol{\beta})\}}. \quad \text{(S1)}$$

By the definition of the KL divergence in (3),

$$d_{KL}(\tilde{f} \parallel f; t_k) = \mathrm{E}_{\tilde{f}}\left[\log\left\{\frac{\tilde{f}(A_k|B_k)}{f(A_k|B_k)}\right\}\right] = \mathrm{E}_{\tilde{f}}\left[\log \tilde{f}(A_k \mid B_k)\right] - \mathrm{E}_{\tilde{f}}[\log f(A_k \mid B_k)]\,. \quad \text{(S2)}$$

Because the expectation is taken under the *external* density $\tilde{f}$, the first term depends only on the fixed external risk scores and is constant in $\boldsymbol{\beta}$; only the cross-entropy term involves $\boldsymbol{\beta}$. This motivates placing the external density in the reference position: under the reverse direction $D_{KL}(f \parallel \tilde{f})$ the expectation weights $f(A_k \mid B_k)$ would themselves depend on $\boldsymbol{\beta}$, and the objective would not reduce to a partial-likelihood form.

Writing $\log f(A_k = j \mid B_k) = g(\mathbf{Z}_j, \boldsymbol{\beta}) - \log\{\sum_i Y_i(t_k)\exp\{g(\mathbf{Z}_i, \boldsymbol{\beta})\}\}$ and using $\sum_j \tilde{f}(A_k = j \mid B_k) = 1$, we obtain

$$\mathrm{E}_{\tilde{f}}[\log f(A_k \mid B_k)] = \sum_{j=1}^{n} \tilde{f}(A_k = j \mid B_k)\, g(\mathbf{Z}_j, \boldsymbol{\beta}) - \log\Big\{\sum_{i=1}^{n} Y_i(t_k)\exp\{g(\mathbf{Z}_i, \boldsymbol{\beta})\}\Big\}. \quad \text{(S3)}$$

Summing (S2) over the K failure times, collecting all $\boldsymbol{\beta}$-free terms into a constant c, and interchanging the order of summation to define the *pseudo-event weight*

$$\tilde{\delta}_i = \sum_{k=1}^{K} \tilde{f}(A_k = i \mid B_k) = \sum_{k=1}^{K} \frac{Y_i(t_k)\exp\{\tilde{g}(\mathbf{Z}_i)\}}{\sum_{j=1}^{n} Y_j(t_k)\exp\{\tilde{g}(\mathbf{Z}_j)\}}, \quad \text{(S4)}$$

the accumulated divergence becomes

$$D_{KL}(\tilde{f} \parallel f) = -\sum_{i=1}^{n} \tilde{\delta}_i \, g(\mathbf{Z}_i, \boldsymbol{\beta}) + \sum_{k=1}^{K} \log\Big\{ \sum_{i=1}^{n} Y_i(t_k) \exp\{g(\mathbf{Z}_i, \boldsymbol{\beta})\} \Big\} + c. \tag{S5}$$

Since the K unique failure times coincide with the observed event times $\{T_i : \delta_i = 1\}$, the risk-set term equals $\sum_{i=1}^{n} \delta_i \log\{\sum_l Y_l(T_i) \exp\{g(\mathbf{Z}_l, \boldsymbol{\beta})\}\}$, which is exactly the risk-set term of (1). Substituting (S5) into (5) and discarding the additive constant $-\eta c$ and the positive factor $(1+\eta)$—neither of which affects the maximizer—gives the closed form

$$\ell_\eta(\boldsymbol{\beta}) \;\propto\; \sum_{i=1}^{n} \left\{ \frac{\delta_i + \eta \, \tilde{\delta}_i}{1+\eta} \, g(\mathbf{Z}_i, \boldsymbol{\beta}) - \delta_i \log\Big[\sum_{l=1}^{n} Y_l(T_i) \exp\{g(\mathbf{Z}_l, \boldsymbol{\beta})\} \Big] \right\}. \tag{S6}$$

Equation (S6) is identical in form to the internal Cox log-partial likelihood (1): the risk sets are unchanged, and the only difference is that the event indicator δ_i is replaced by the convex combination $(\delta_i + \eta \, \tilde{\delta}_i)/(1+\eta)$ of the observed event indicator δ_i and the external pseudo-event weight $\tilde{\delta}_i$. Integrating the external information therefore amounts to fitting a single Cox model with a modified pseudo-outcome, at the same computational cost as an ordinary partial-likelihood fit. The weight η interpolates between the internal-only fit ($\eta = 0$, where the pseudo-outcome reduces to δ_i) and full reliance on the external ranking ($\eta \to \infty$).

S1.2 Integrating multiple external sources

The framework extends to *multiple* external sources, and the cross-validation that selects the integration weight η also gives a natural criterion for ranking and combining them. For each candidate external model, the cross-validated C-index traces a curve along the η-path whose height quantifies how much that source improves on the internal model (Figure S1). A strongly informative source reaches a high cross-validated optimum at a sizeable η, whereas an uninformative or adversarial source peaks at $\eta \approx 0$ and is effectively ignored. We integrate sources *sequentially*: beginning with the source of highest cross-validated benefit, we add the next-best source at each step and re-select η by cross-validation. Because a non-beneficial source drives its own weight toward 0—the robustness to negative transfer illustrated in Section S2.1—adding further, weaker sources does not degrade the integrated model.

S2 Supplementary Results

S2.1 Benchmarking against privacy-preserving transfer-learning methods

We compared `survkl` with two other privacy-preserving (summary-level) transfer-learning methods: Trans-Cox (Li et al., 2023) and Mahalanobis-distance-based transfer learning (MDTL), the latter evaluated both with an identity (L_2) weight and with the external Fisher information matrix as the Mahalanobis weight. Using the six-covariate simulation design of the main text (Example Usage), we compared an informative external source (no population heterogeneity) with two negative-transfer extremes: an uninformative *Null* source (external coefficients set to zero) and an adversarial *Harmful* source (sign-flipped coefficients), over 500 repeated train–test splits (internal $n = 100$, test $n = 2{,}000$). Table S1 reports the C-index, the negative partial log-likelihood (loss), the Integrated Brier Score (IBS), and the integrated time-dependent AUC (itdAUC).

When the external source is informative, all integration methods attain comparable accuracy and `survkl` matches the Mahalanobis-distance methods throughout. Under the *Null* and *Harmful*

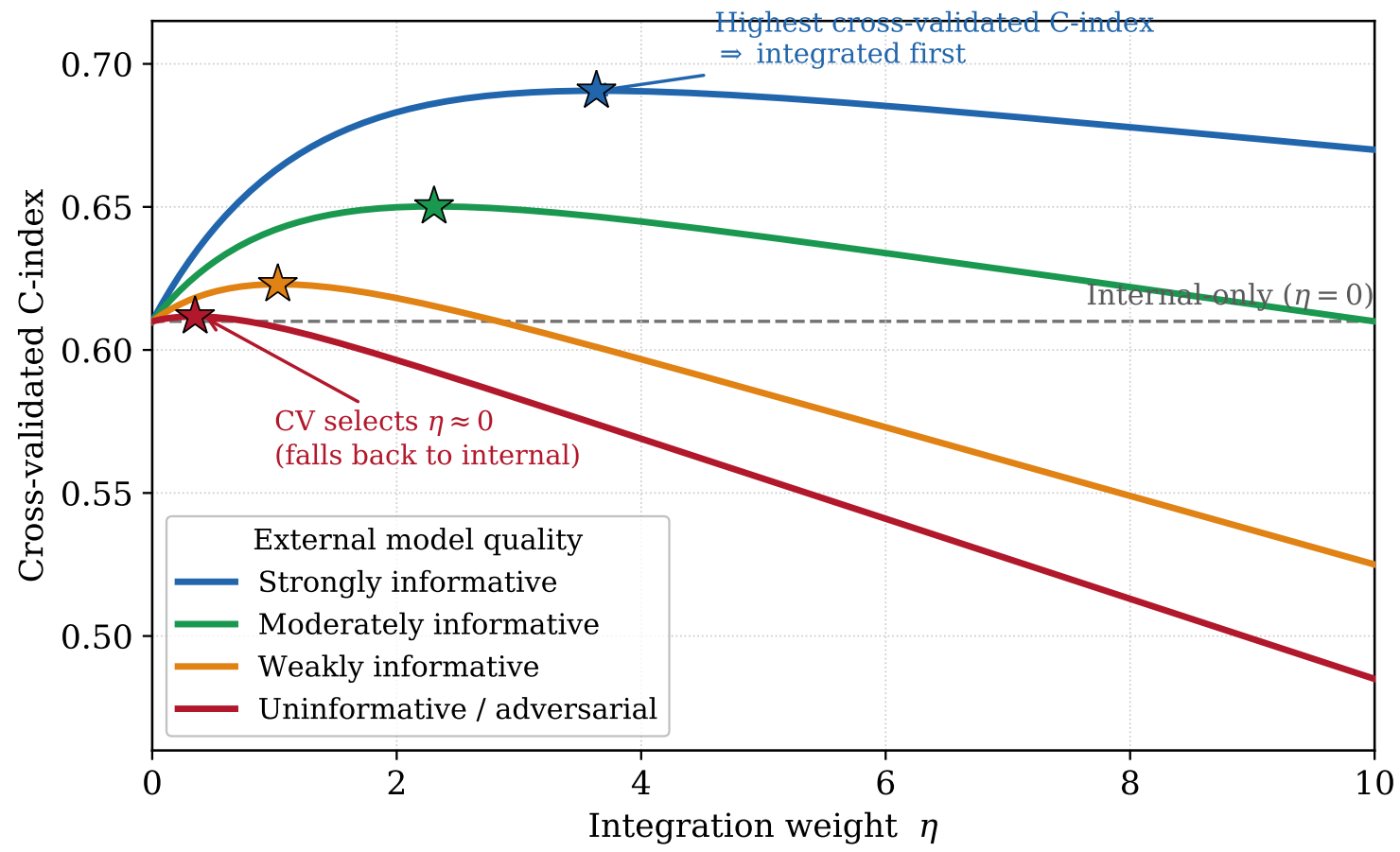

Figure S1: Illustrative behaviour of the cross-validated C-index along the integration-weight (η) path for external sources of differing quality. At $\eta = 0$ every curve coincides with the internal-only model (dashed line); increasing η borrows more heavily from the external source. A strongly informative source (blue) rises to a high cross-validated optimum and degrades only slowly, whereas progressively weaker sources peak lower and earlier; an uninformative or adversarial source (red) barely improves on the internal model and its cross-validated optimum sits at $\eta \approx 0$, so it is effectively ignored. Stars mark the cross-validation–selected weight for each source. With multiple external sources, `survkl` integrates them sequentially, starting from the source with the highest cross-validated C-index.

sources, `survkl` holds discrimination at the internal-only level (C-index ≈ 0.61), whereas the external model alone collapses to 0.431 and 0.351, respectively, and `survkl` never falls below the internal model on any metric. Among these methods `survkl` requires the least external information—only a risk score or coefficient vector, compared with Trans-Cox's additional need for the external baseline hazard and MDTL's need for the external second-order information—and it converged in every replication, whereas Trans-Cox produced non-finite estimates in 40.0% of the no-heterogeneity replications (and none under the *Null* and *Harmful* sources); its summaries are computed over the converged replications only.

S2.2 Additional predictive metrics on the multiple myeloma data

For the multiple myeloma (MM) application of the main text (Example Usage), we report two further metrics—IBS and itdAUC—alongside the C-index on the held-out TT2 test data, comparing the internal-only LASSO, external-only LASSO, KL-divergence-based integrated LASSO, and stacked models over 500 repeated random splits (Table S2). The integrated model attains the best discrimination, with the highest C-index (0.681) and itdAUC (0.659), ahead of the internal-only (0.592; 0.578), external-only (0.666; 0.619), and stacked (0.620; 0.600) models, and with markedly lower variability than the internal-only model. Its calibration matches that of the stacked model (IBS 0.101 versus 0.099); both, together with the external-only model, improve substantially on the internal-only model (IBS 0.117). The *stacked* model pools the internal and external individual-level data without any privacy constraint, yet it does not outperform the privacy-preserving integrated model. Across the 500 splits, the integrated model's per-split gain in C-index over the internal-only, external-only, and stacked models is significant under both a paired t-test and a Wilcoxon signed-rank test ($p < 0.001$ in every comparison).

S2.3 Computational runtime

The closed form (S6) implies that a single integrated fit costs essentially the same as one ordinary Cox fit, and the following benchmarks confirm that the cost grows smoothly with the training sample size n and the number of covariates p. On the simulated Cox problem, we recorded the wall-clock time of a single integrated fit under one fixed integration weight η over a 100-value λ-path, as a function of n (at fixed $p = 2{,}000$) and of p (at fixed $n = 300$), over 100 replications per setting (Table S3). A single fixed-η fit takes well under a second on small problems and about 12 seconds even at $p = 20{,}000$, the scale of the MM application. On the MM microarray data ($p \approx 20{,}500$ genes), the full cross-validation over 100 candidate weights η completes in 14.5 minutes, and a single-η fit takes approximately 8.7 seconds—comparable to fitting a standard penalized Cox model without integration using `glmnet` (Simon et al., 2011) ($\approx$ 7.6 s) or `grpreg` (Breheny and Huang, 2015) ($\approx$ 9.1 s).

References

Breheny P, Huang J. Group descent algorithms for nonconvex penalized linear and logistic regression models with grouped predictors. *Statistics and Computing* 2015;25:173–187.

Li Z, Shen Y, Ning J. Accommodating time-varying heterogeneity in risk estimation under the Cox model: a transfer learning approach. *Journal of the American Statistical Association* 2023;118:2276–2287.

Simon N, Friedman J, Hastie T *et al*. Regularization paths for Cox's proportional hazards model via coordinate descent. *Journal of Statistical Software* 2011;39:1.

Model	C-index ↑		Loss ↓		IBS ↓		itdAUC ↑	
	Mean	SD	Mean	SD	Mean	SD	Mean	SD
No heterogeneity (informative external)								
Internal	0.615	0.026	1.996	0.084	0.161	0.010	0.637	0.036
External	0.648	0.014	1.951	0.074	0.149	0.005	0.681	0.018
survkl (proposed)	0.645	0.024	1.960	0.077	0.151	0.007	0.669	0.028
Trans-Cox	0.624	0.025	1.974	0.080	0.156	0.008	0.650	0.032
MDTL (L_2)	0.640	0.024	1.960	0.078	0.152	0.008	0.668	0.030
MDTL (Mahalanobis)	0.642	0.031	1.966	0.084	0.153	0.010	0.667	0.035
Null (uninformative) external								
Internal	0.614	0.029	1.996	0.084	0.161	0.010	0.637	0.036
External	0.431	0.013	1.999	0.076	0.167	0.005	0.417	0.020
survkl (proposed)	0.613	0.028	1.984	0.079	0.159	0.008	0.636	0.048
Trans-Cox	0.612	0.031	1.977	0.076	0.157	0.008	0.635	0.038
MDTL (L_2)	0.602	0.044	1.983	0.079	0.159	0.008	0.623	0.054
MDTL (Mahalanobis)	0.604	0.051	1.997	0.084	0.162	0.010	0.625	0.063
Harmful (adversarial) external								
Internal	0.614	0.029	1.996	0.084	0.161	0.010	0.637	0.036
External	0.351	0.013	2.143	0.081	0.217	0.007	0.320	0.018
survkl (proposed)	0.612	0.034	1.996	0.084	0.162	0.012	0.623	0.067
Trans-Cox	0.598	0.035	1.977	0.076	0.158	0.007	0.618	0.043
MDTL (L_2)	0.601	0.056	1.994	0.083	0.162	0.013	0.621	0.068
MDTL (Mahalanobis)	0.606	0.054	2.001	0.087	0.163	0.014	0.628	0.066

Table S1: Predictive performance of privacy-preserving (summary-level) transfer-learning methods over 500 repeated train–test splits (internal $n = 100$, test $n = 2{,}000$) under three external-source scenarios: *No heterogeneity* (matched covariate distribution and correctly specified coefficients), *Null* (external coefficients set to zero), and *Harmful* (sign-flipped coefficients). *Internal* uses no external information; *External* applies the external model alone; *survkl* is the proposed KL-divergence-based integration; *Trans-Cox* and *MDTL* are summary-level transfer-learning benchmarks, MDTL evaluated with an identity (L_2) weight and with the external Fisher information matrix (Mahalanobis) as the weight. Loss is the negative partial log-likelihood; IBS is the Integrated Brier Score; itdAUC is the integrated time-dependent AUC. Higher is better for the C-index and itdAUC (↑); lower is better for loss and IBS (↓). All methods converged in every replication except Trans-Cox, which produced non-finite estimates in 40.0% of the no-heterogeneity replications (none under *Null*/*Harmful*); its summaries use the converged replications only.

Model	C-index ↑		IBS ↓		itdAUC ↑	
	Mean	SD	Mean	SD	Mean	SD
Internal	0.592	0.049	0.117	0.020	0.578	0.065
External	0.666	0.011	0.099	0.002	0.619	0.008
Integrated	0.681	0.014	0.101	0.002	0.659	0.026
Stacked	0.620	0.014	0.099	0.001	0.600	0.022

Table S2: Predictive performance of the internal-only LASSO, external-only LASSO, KL-divergence-based integrated LASSO, and stacked models on the held-out TT2 test data over 500 repeated random splits. The *stacked* model pools the internal and external individual-level data without a privacy constraint. IBS is the Integrated Brier Score; itdAUC is the integrated time-dependent AUC. Higher is better for the C-index and itdAUC (↑); lower is better for IBS (↓).

Setting	Median (s)	IQR (s)
(A) Scaling with sample size n (fixed $p = 2{,}000$)		
$n = 100$	0.81	[0.78, 0.83]
$n = 200$	0.98	[0.95, 1.03]
$n = 400$	1.30	[1.27, 1.38]
$n = 800$	1.88	[1.76, 2.23]
$n = 1{,}600$	2.70	[2.60, 2.83]
$n = 3{,}200$	4.12	[4.00, 4.26]
(B) Scaling with number of variables p (fixed $n = 300$)		
$p = 100$	0.07	[0.06, 0.07]
$p = 500$	0.37	[0.35, 0.39]
$p = 1{,}000$	0.65	[0.62, 0.68]
$p = 2{,}000$	1.12	[1.08, 1.16]
$p = 5{,}000$	2.57	[2.49, 2.67]
$p = 10{,}000$	5.64	[5.44, 5.90]
$p = 20{,}000$	11.75	[11.42, 12.33]

Table S3: Wall-clock time for a single integrated fit of `survkl` under one fixed integration weight η over a full 100-value λ-path, reported as the median and interquartile range (IQR) over 100 replications. Panel (A) varies the training sample size n at fixed $p = 2{,}000$; Panel (B) varies the number of covariates p at fixed $n = 300$.
